# Development of a highly sensitive method to detect translational infidelity

**DOI:** 10.1093/biomethods/bpaf008

**Published:** 2025-01-25

**Authors:** Max Hartmann, Lisa Neher, Benjamin Grupp, Zhouli Cao, Chloe Chiew, Sebastian Iben

**Affiliations:** Department of Dermatology and Allergic Diseases, Ulm University, 89081 Ulm, Germany; Department of Dermatology and Allergic Diseases, Ulm University, 89081 Ulm, Germany; Department of Molecular Genetics, Ulm University, 89081 Ulm, Germany; Department of Dermatology and Allergic Diseases, Ulm University, 89081 Ulm, Germany; Department of Dermatology and Allergic Diseases, Ulm University, 89081 Ulm, Germany; Department of Dermatology and Allergic Diseases, Ulm University, 89081 Ulm, Germany

**Keywords:** ribosome, translational fidelity, nanoluciferase

## Abstract

Protein homeostasis (proteostasis) is the balance of protein synthesis, protein maintenance, and degradation. Loss of proteostasis contributes to the aging process and characterizes neurodegenerative diseases. It is well established that the processes of protein maintenance and degradation are declining with aging; however, the contribution of a declining quality of protein synthesis to the loss of proteostasis is less well understood. In fact, protein synthesis at the ribosome is an error-prone process and challenges the cell with misfolded proteins. Here, we present the development of a highly sensitive and reproducible reporter assay for the detection of translational errors and the measurement of translational fidelity. Using Nano-luciferase, an enzyme 3 times smaller and 50 times more sensitive than the hitherto used Firefly-luciferase, we introduced stop-codon and amino-acid exchanges that inactivate the enzyme. Erroneous re-activation of luciferase activity indicates ribosomal inaccuracy and translational infidelity. This highly sensitive and reproducible method has broad applications for studying the molecular mechanisms underlying diseases associated with defective protein synthesis and can be used for drug screening to modulate translational fidelity.

## Introduction

Protein synthesis by the ribosome is an error-prone process. Whereas transcription of mRNA by human RNA polymerase II yields 4.7 × 10^−6^ errors per base pair [1] and the charging of amino acids on the cognate tRNA has an error frequency of 1 × 10^−6^ [2], ribosomal protein translation has an estimated rate of 1 × 10^−3^ to 10^−4^ errors per translated mRNA codon [3, 4]. An error rate of 5 × 10^−4^ by protein synthesis leads to nearly one-fifth of the proteome (18%) (proteins that are expressed from an average length of ∼400 codons per gene) containing at least one missense substitution [5].

Translational fidelity coevolved with longevity, since long-living rodents display higher translational accuracy than short-living rodent species [6]. Increasing the error rate of the ribosome can shorten the lifespan, whilst a decreased error rate prolongates the lifespan of model organisms [7–9].

How translational fidelity is regulated in human aging and disease is poorly defined. Premature aging diseases, syndromes that display an accelerated aging phenotype like Cockayne syndrome and trichothiodystrophy, have been shown to suffer from a general loss of proteostasis because of defective ribosomal biogenesis and subsequent elevated ribosomal error rate [10–12]. This pathomechanism was recently demonstrated in the late-onset neurodegenerative disease Huntington where mutant huntingtin disturbs ribosomal biogenesis leading to impaired translational fidelity and whole proteome instability [13]. In all neurodegenerative diseases of the aging body, protein aggregation is a symptom, if not a driver of the pathogenesis. However, where the aggregation-prone proteins are coming from is still a matter of debate. Our group focuses on the hypothesis that faulty translation at the ribosome could contribute to, or cause the failure of the clearance systems and fuel aggregation [14].

To investigate ribosomal error rate luciferase-based assays were developed, introducing defined mutations in sites that are crucial for enzymatic function [4, 15]. These mutations are either near-cognate base mismatches (one position in the triplet) or non-cognate (two or more base mismatches) codons or frameshift mutations leading to ±11 frameshifts to study for example programmed ribosomal frameshifts [16]. Reactivation of luciferase by misincorporating the “right” amino acid indicates erroneous translation and overall luciferase activity mirrors error frequencies. Luciferase-based assays can be performed in living cells by transfecting reporter plasmids, reporter mRNA, with cytosolic extracts or even with purified ribosomes reconstituted with ribosome-depleted extracts from HeLa or HEK cells [17, 18] or rabbit-reticulocyte lysates [19]. While transfection experiments and experiments with cytosolic extracts cannot discriminate between influences of tRNA mischarging or translation initiation/elongation factors on translational fidelity, reconstituted systems with purified ribosomes assess specifically the error rate of the ribosomes. In translational fidelity experiments, the sensitivity of Firefly luciferase is the limiting factor. To overcome this hurdle we developed a more sensitive, highly reproducible, and stable method to measure errors in translation. The Nano-luciferase (NanoLuc) provides the ideal template since it’s a small protein and the activity exceeds Firefly-luciferase by 100 times [20].

## Materials and methods

### Luciferase comparison

To compare the activity of NanoLuc with Firefly luciferase, we transfected equal absolute amounts of WT plasmids (500 ng) in 450 000 FF95 cells. Firefly plasmid (5256 bp) has 1.5 times more base pairs than the NanoLuc plasmid (3579 bp). RT-qPCR showed a 2.1 times higher expression level of the NanoLuc. Dividing the mRNA level by the plasmid ratio, we obtained a 1.4 higher transcription rate of the NanoLuc. Dividing the ratio of NanoLuc to Firefly activity with the 1.4 transcription rate, we calculated 50 times higher NanoLuc activity per expressed mRNA than Firefly. This result suggests that NanoLuc assays are 50 times more sensitive than Firefly luciferase-based assays.

### Plasmid construction and preparation

To generate the NanoLuc constructs to measure ribosomal error rate, the pNL1.1.PGK [Nluc/PGK] vector (Promega, N1441) was modified. Several mutations were induced using the Phusion Site-Directed Mutagenesis Kit (Thermo Fisher, F541). Tyrosine (TAC) at position 18 was changed into a TAA stop codon (forward primer: 5′-GAC AGC CGG CTA AAA CCT GGA CCA-3′; reverse primer: 5′-TGT CGC CAG TCC CCA ACG AAA TCT TC-3′) and a TGA stop codon (forward primer: 5′-GAC AGC CGG CTG AAA CCT GGA CCA-3′, reverse primer: 5′-TGT CGC CAG TCC CCA ACG AAA TCT TC-3′). For amino acid substitution, the arginine (CGG) at position 162 was mutated into a near-cognate serine (AGC) (forward primer: 5′-GAC CGG CTG GAG CCT GTG CGA AC-3′, reverse primer: 5′-ACT CCG TTG ATG GTT ACT CGG AAC AGC AG-3′) and a non-cognate serine (TCT) (forward primer: 5′-GAC CGG CTG GTC TCT GTG CGA AC-3′, reverse primer: 5′-ACT CCG TTG ATG GTT ACT CGG AAC AGC AG-3′). The near-cognate serine has a two-base pair overlap with three of the arginine codons, whereas the non-cognate serine has a maximum one-base pair overlap with any arginine codon (Supplementary Fig. S1). The altered plasmids were transformed into competent *E.coli* using heat shock and plated onto Agar plates containing the selection antibiotic ampicillin. Single colonies were inoculated in 5 ml LB-media (including ampicillin) overnight at 37°C. The plasmid was isolated using the GeneJET Plasmid-Miniprep-Kit (Thermo Fisher, K0503) and sent for Sanger sequencing (Micorsynth) to confirm the introduced mutation. To obtain high amounts of plasmids, the QIAGEN Plasmid Maxi Kit (QIAGEN, 12162) was used.

### Workflow to measure translational fidelity using electroporation

To establish this method, we used different adherent (primary foreskin fibroblasts (FF95) and HEK293T (RRID: CVCL_0063) cell lines). Cells should be at 70%–80% confluency at the time point of harvest. After detaching, the cells must be counted to ensure a reproducible number of cells for each experiment. In this study, we used 450 000 cells for every technical triplicate. Depending on the transfection efficiency of the cell line, we co-transfected 3–5 µg of NanoLuc plasmid and 0.3–0.5 µg of Firefly plasmid (pGL3/SV40, Promega E1751) via electroporation. The NanoLuc activity represents the translation error, whereas the Firefly activity is used to normalize the transfection efficiency. We used the Neon™ Transfection System (Invitrogen, MPK5000) for electroporation. The subsequent steps are time-critical and should be performed as fast as possible to avoid cell stress and obtain high transfection efficiencies. Pelleted 450 000 cells were resuspended in 8 µl of SB-buffer (250 mM sucrose and 1 mM MgCl2 in 1× DPBS) and transferred to the plasmid mix (NanoLuc- plus Firefly-plasmid in a maximal volume of 4 µl). After careful mixing with a 10 µl Neon™ tip, the solution was taken up avoiding any air bubbles within the tip and electroporated. Preset transfection conditions used were 1100 V, 20 ms, and 2 pulses. The transfected cells were immediately transferred to a tube containing 150 µl prewarmed cell culture medium. After each transfection, 50 µl were distributed into 3 wells of a white Nunc^®^ MicroWell™ 96-well polystyrene plate (Sigma-Aldrich, P8616). Twenty-four hours after transfection, the Nano-Glo^®^ Dual-Luciferase^®^ Reporter Assay System (Promega, N1620) reagents in combination with the Varioskan LUX Multimode Microplate Reader (Thermo Fisher Scientific, N16045) were used according to manufacturer’s protocol to detect the bioluminescence. The calculated ratio of the NanoLuc signal divided through the Firefly signal displays the ribosomal error rate.

## Results and discussion

Although protein synthesis by the ribosome is an error-prone process, detection and quantification of these errors are challenging. Firefly-luciferase is a large protein of 550 amino acids. NanoLuc was developed using a small luciferase subunit (19 kDa) from the deep sea shrimp *Oplophorus gracilirostris* and displays a significantly higher specific activity than Firefly and Renilla luciferases [20].

**Figure 1.**
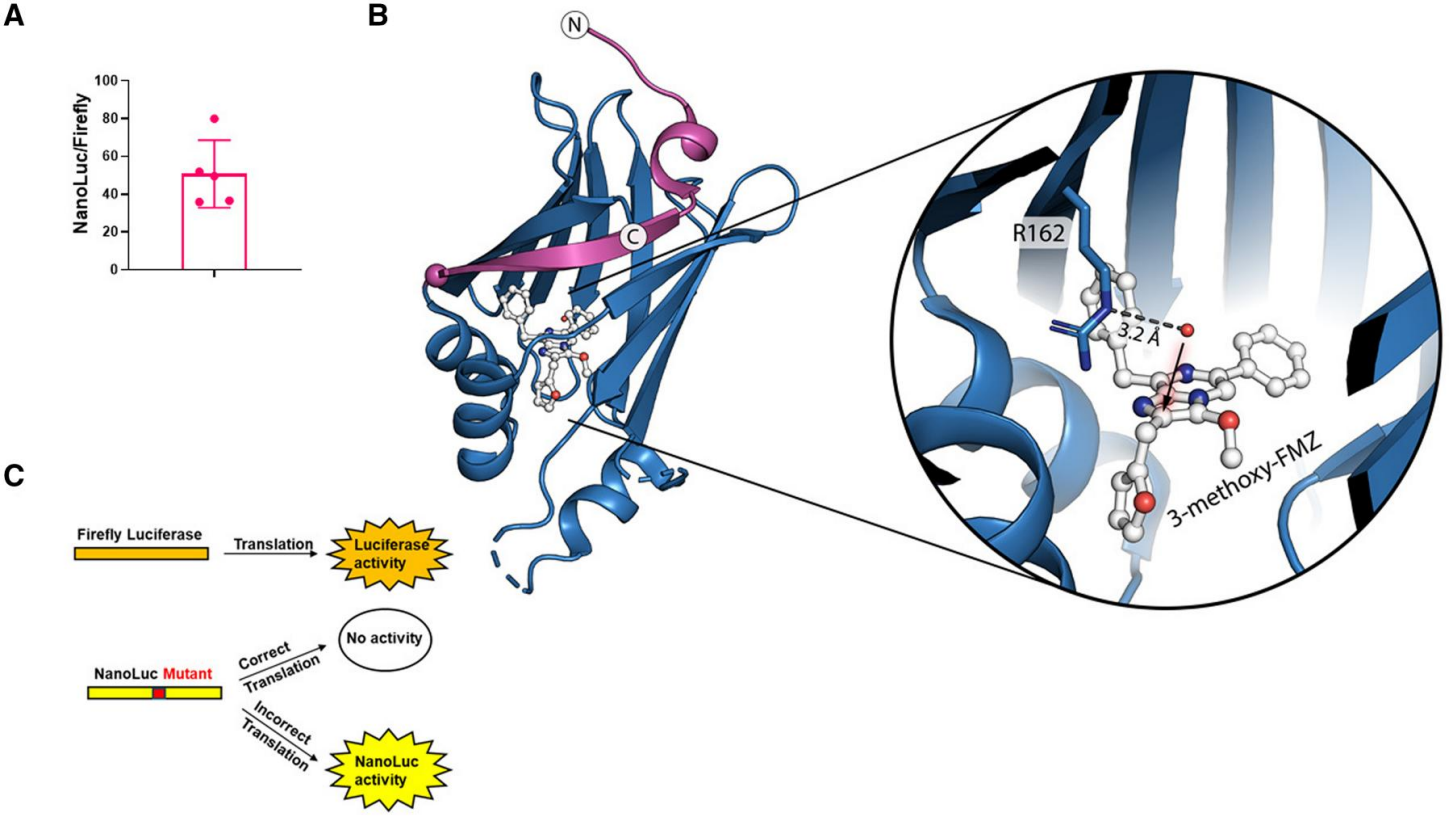
Activity-ratio between NanoLuc and Firefly luciferase. **(A)** Equal amounts of NanoLuc or Firefly luciferase encoding plasmids were co-transfected in fibroblasts of healthy young donors and luciferase activities were detected 24 h later. **(B)** (Left) The NanoLuc-structure is displayed including the substrate analog 3-methoxy-furimazine (FMZ) substrate. Zooming in the active center of the enzyme reveals an important role of the arginine at position 162. This arginine helps in positioning of free dioxygen (co-substrate) and is responsible for the protonation of FMZ in the process of the NanoLuc catalysis. The arrow indicates the proposed position of the radical-mediated reaction between the coordinated dioxygen molecule (here: water molecule) and FMZ. The structure and reaction mechanism indicate that R162 is crucial for the NanoLuc activity. Magenta labeling indicates the NanoLuc peptide in front of the introduced stop codons. **(C)** Scheme of the translational fidelity experiments.

To verify that this difference becomes apparent in transfection assays, we transfected equal amounts of NanoLuc and Firefly-luciferase encoding plasmids into fibroblasts and, taking different transcription efficiency into account, measured mRNA expression and specific activity 24 h later. In fact, NanoLuc displayed specific luminescence −50 fold brighter than Firefly-luciferase (Fig. 1A). Encouraged by this result we decided to introduce different mutations into the NanoLuc coding region of the plasmid to obtain mutant reporter plasmids. First, we introduced stop-codons at the amino acid position 18, changing the tyrosine-encoding codon TAC by one base to the near-cognate stop codon TAA and by two bases to the non-cognate stop codon TGA When correctly translated, the stop codons lead to an interrupted translation, resulting in a nascent amino-acid chain of 17 aa (magenta part of structure, Fig. 1B). This truncated N-terminal part of NanoLuc is enzymatically inactive. To detect amino-acid exchanges we decided to mutate the arginine (R) at position 162, because it is likely involved in the coordination of the dioxygen co-substrate, is important for the protonation of the activated catalytic intermediate and thus plays a central role in the catalytic activity [21]. We constructed two serine codons substituting arginine (R) 162 to serine (S) intending to inactivate luciferase activity. The non-cognate (TCT) serine has two different base pairs compared to any arginine codon, whereas the near cognate (AGC) has two base pairs overlapping with three codons typical for arginine (Supplementary Fig. S1). The non-cognate serine has a lower chance of being resubstituted to the original arginine thereby acting as a control for the inactivation of the NanoLuc.

**Figure 2.**
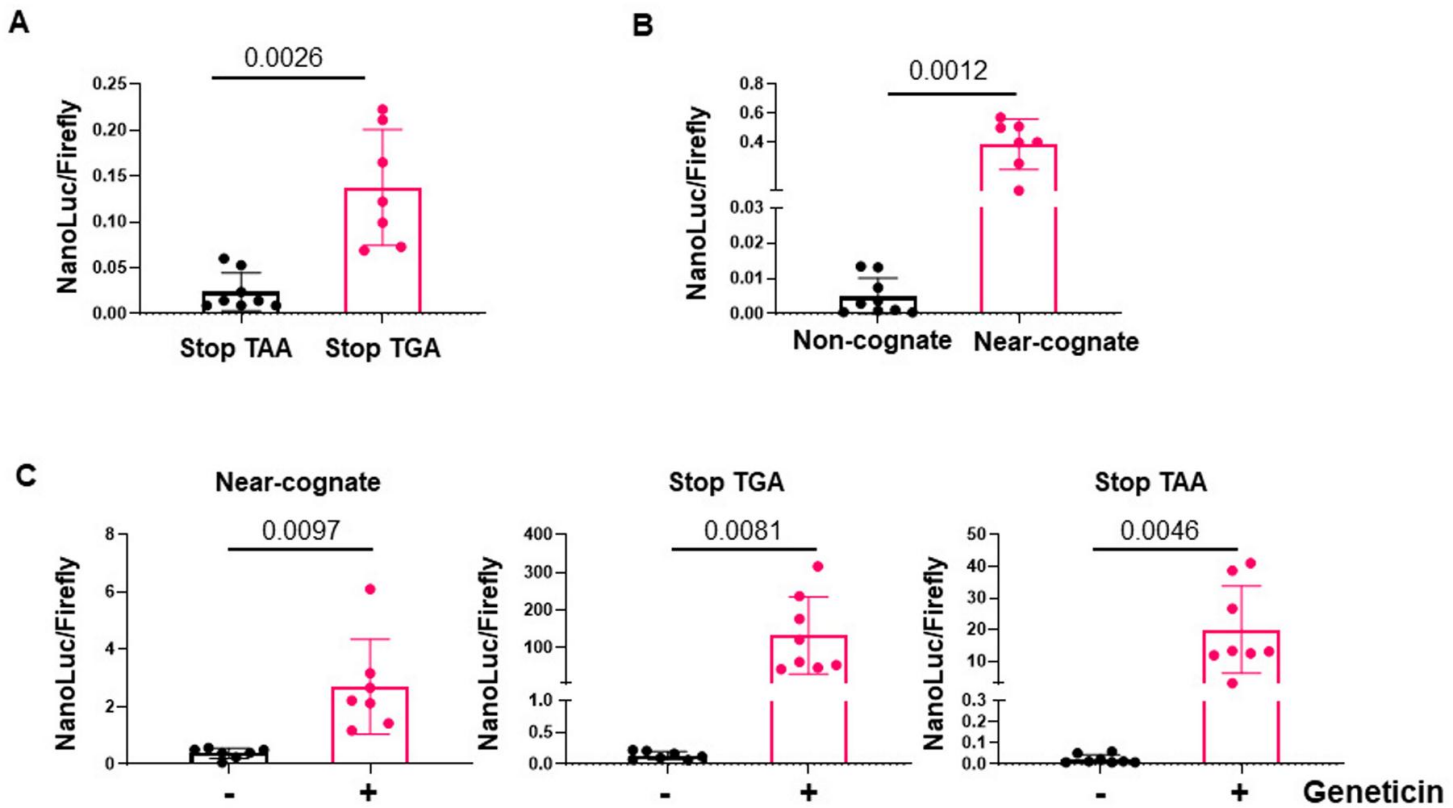
Translational fidelity experiments with NanoLuc constructs **(A)** Stop codon-readthrough in fibroblasts. Stop codons show a different rate of readthrough depending on the codon. The y-axis displays the readthrough by the ratio of NanoLuc to firefly. Each dot represents an independent experiment (biological replicate), the mean of three technical replicates. For statistical analysis using biological replicates, an unpaired t-test with Welch’s correction was utilized. The P-value is shown in the figure. (**B**) Translational fidelity experiments with the mutant codons at amino-acid position 162 (R162S) demonstrate that the non-cognate construct is not reactivated, whereas the near-cognate construct yields a significant amount of amino-acid exchanges re-activating NanoLuc activity. Each dot represents an independent experiment (biological replicate), the mean of three technical replicates. Statistical analysis using biological replicates was performed using an unpaired *t*-test with Welch’s correction, *P*-values are shown in the figure (**C**). Geneticin treatment enhances translational infidelity, the error rate of translation. Cells were treated 24 h with geneticin after transfection. Statistical analysis using biological replicates was performed using an unpaired *t*-test with Welch’s correction, *P*-values are shown in the figure.

After mutation verification by sequencing (Supplementary Fig. S1), we co-transfected first the stop-codon constructs into fibroblasts from healthy young donors with a plasmid encoding Firefly-luciferase as a transfection control (Fig. 2A). Interestingly the non-cognate stop codon was read-through at a much higher rate than the near-cognate construct indicating different strengths of the stop signals as previously reported [22]. Additionally, tyrosine 18 is part of the outer structure of the NanoLuc, far from the active center and it is tempting to speculate that not only a misincorporation of the original tyrosine but a random incorporation of any amino acid can restore the NanoLuc activity.

Next, we tested the mutant constructs with amino-acid exchanges experimentally by co-transfection into low-passage skin fibroblasts (Fig. 2B). Indeed, NanoLuc activity was barely detectable from the non-cognate construct suggesting that arginine replacement by serine inactivates the NanoLuc. In contrast, transfection of the near-cognate plasmid yielded a significant NanoLuc activity, indicating the erroneous incorporation of arginine at position 162 thereby reflecting the innate error rate of human fibroblasts. Translational errors can be induced by the use of the aminoglycoside antibiotic geneticin. Geneticin interacts with prokaryotic and eukaryotic ribosomal RNA and affects the decoding center of the small ribosomal subunit that differ in critical residues in prokaryotes and eukaryotes [23] thus providing the base of selectivity. Aminoglycoside antibiotics are used to induce translational errors experimentally [7, 24, 25] and we tested if Geneticin re-activates the mutant NanoLuc reporters in transfection experiments with human fibroblasts. As demonstrated in Fig. 2C Geneticin significantly enhanced the misincorporation of the “right,” activating amino acid in the near-cognate R162S mutant and highly stimulated the readthrough of the different stop-codon constructs, indicating that these mutant NanoLuc plasmids measure translational errors.

**Figure 3.**
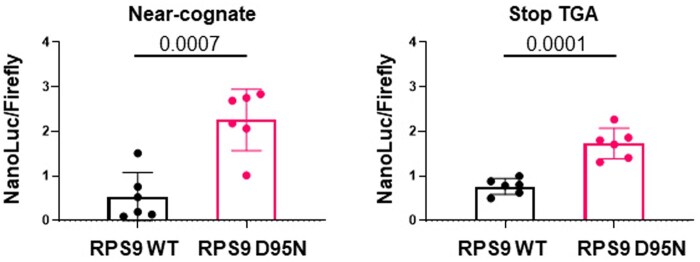
Translational fidelity experiments in the presence of error-prone ribosomes. Overexpression of mutant (D95N), but not wildtype RPS9 induces an elevation of amino-acid exchanges (Near-cognate) or stop-codon readthrough (Stop TGA) events. For statistical analysis of the biological replicates an unpaired t-test was performed, the p-values are shown in the figure.

To further verify these results we established a HEK293T cell-based model that modifies the translational fidelity of the ribosome by overexpressing the D95N mutant of the small ribosomal subunit protein 9 (RPS9 D95N) (Supplementary Fig. S2). This mutation induces premature aging and Alzheimer-like symptoms in mice [7, 8], by weakening the interaction of RPS9 and RPS2 followed by a reduced discrimination of near-cognate tRNAs. Interestingly, overexpression of the mutant, but not of wild-type RPS9, induced a significant re-activation of the transfected mutant NanoLuc reporters (Fig. 3). Assuming that the overexpressed RPS9 proteins integrate functionally into the host’s ribosomes as shown by Shcherbakov [7] this result indicates that the NanoLuc reporters measure translational errors. Taken together these results provide evidence for the successful generation of a highly sensitive assay system to detect translational errors.

## Conclusion

We were able to develop a highly sensitive method to measure errors in translation in cell systems. This method has been already tested in several different cell lines including for example primarily, immortalized fibroblasts, HEK293T cells, neuroblastoma cell lines, and mouse fibroblasts. We are confident that this method is suitable for any transfectable cell line that yields a sufficient amount of cells. Furthermore, translational fidelity can be measured in inhibitor/drug screening experiments to investigate influences on translation. The method can be established in any laboratory that has a basic level of expertise in the maintenance and transfection of cells.

## Supplementary Material

bpaf008_Supplementary_Data

## Data Availability

Data available in supplementary material.

## References

[1] Chung C , Verheijen BM, Navapanich Z et al Evolutionary conservation of the fidelity of transcription. Nat Commun 2023;14:1547. 36941254 10.1038/s41467-023-36525-w PMC10027832

[2] Soll D. The accuracy of aminoacylation—ensuring the fidelity of the genetic code. Experientia 1990;46:1089–96. 10.1007/BF01936918 2253707

[3] Ogle JM , Ramakrishnan V. Structural insights into translational fidelity. Annu Rev Biochem 2005;74:129–77. 15952884 10.1146/annurev.biochem.74.061903.155440

[4] Kramer EB , Farabaugh PJ. The frequency of translational misreading errors in E. coli is largely determined by tRNA competition. RNA 2007;13:87–96. 17095544 10.1261/rna.294907 PMC1705757

[5] Drummond DA , Wilke CO. Mistranslation-induced protein misfolding as a dominant constraint on coding-sequence evolution. Cell 2008;134:341–52. 18662548 10.1016/j.cell.2008.05.042 PMC2696314

[6] Ke Z , Mallik P, Johnson AB et al Translation fidelity coevolves with longevity. Aging Cell 2017;16:988–93. 28707419 10.1111/acel.12628 PMC5595694

[7] Shcherbakov D , Nigri M, Akbergenov R et al Premature aging in mice with error-prone protein synthesis. Sci Adv 2022;8:eabl9051. 35235349 10.1126/sciadv.abl9051 PMC8890705

[8] Brilkova M , Nigri M, Kumar HS et al Error-prone protein synthesis recapitulates early symptoms of Alzheimer disease in aging mice. Cell Rep 2022;40:111433. 36170830 10.1016/j.celrep.2022.111433

[9] Martinez-Miguel VE , Lujan C, Espie-Caullet T et al Increased fidelity of protein synthesis extends lifespan. Cell Metab 2021;33:2288–300 e12. 34525330 10.1016/j.cmet.2021.08.017 PMC8570412

[10] Alupei MC , Maity P, Esser PR et al Loss of Proteostasis Is a Pathomechanism in Cockayne Syndrome. Cell Rep 2018;23:1612–9. 29742419 10.1016/j.celrep.2018.04.041

[11] Khalid F , Phan T, Qiang M et al TFIIH mutations can impact on translational fidelity of the ribosome. Hum Mol Genet 2023;32:1102–13. 36308430 10.1093/hmg/ddac268 PMC10026254

[12] Phan T , Maity P, Ludwig C et al Nucleolar TFIIE plays a role in ribosomal biogenesis and performance. Nucleic Acids Res 2021;49:11197–210. 34581812 10.1093/nar/gkab866 PMC8565312

[13] Wagner M , Zhu G, Khalid F et al General loss of proteostasis links Huntington disease to Cockayne syndrome. Neurobiol Dis 2024;201:106668. 39284372 10.1016/j.nbd.2024.106668

[14] Iben S. To aggregate or not to aggregate—is it a matter of the ribosome? Bioessays 2023;45:e2200230. 37194995 10.1002/bies.202200230

[15] Salas-Marco J , Bedwell DM. Discrimination between defects in elongation fidelity and termination efficiency provides mechanistic insights into translational readthrough. J Mol Biol 2005;348:801–15. 15843014 10.1016/j.jmb.2005.03.025

[16] Dinman JD. Mechanisms and implications of programmed translational frameshifting. Wiley Interdiscip Rev RNA 2012;3:661–73. 22715123 10.1002/wrna.1126 PMC3419312

[17] Gonskikh Y , Pecoraro V, Polacek N. Mammalian in vitro translation systems. Methods Mol Biol 2022;2428:101–11. 35171476 10.1007/978-1-0716-1975-9_7

[18] Aleksashin NA , Chang ST, Cate JHD. A highly efficient human cell-free translation system. RNA 2023;29:1960–72. 37793791 10.1261/rna.079825.123 PMC10653386

[19] Penzo M , Carnicelli D, Montanaro L, Brigotti M. A reconstituted cell-free assay for the evaluation of the intrinsic activity of purified human ribosomes. Nat Protoc 2016;11:1309–25. 27336708 10.1038/nprot.2016.072

[20] Hall MP , Unch J, Binkowski BF et al Engineered luciferase reporter from a deep sea shrimp utilizing a novel imidazopyrazinone substrate. ACS Chem Biol 2012;7:1848–57. 22894855 10.1021/cb3002478 PMC3501149

[21] Nemergut M , Pluskal D, Horackova J et al Illuminating the mechanism and allosteric behavior of NanoLuc luciferase. Nat Commun 2023;14:7864. 38030625 10.1038/s41467-023-43403-y PMC10687086

[22] Palma M , Lejeune F. Deciphering the molecular mechanism of stop codon readthrough. Biol Rev Camb Philos Soc 2021;96:310–29. 33089614 10.1111/brv.12657

[23] Prokhorova I , Altman RB, Djumagulov M et al Aminoglycoside interactions and impacts on the eukaryotic ribosome. Proc Natl Acad Sci U S A 2017;114:E10899–E10908. 29208708 10.1073/pnas.1715501114 PMC5754804

[24] Wohlgemuth I , Garofalo R, Samatova E et al Translation error clusters induced by aminoglycoside antibiotics. Nat Commun 2021;12:1830. 33758186 10.1038/s41467-021-21942-6 PMC7987974

[25] Shcherbakov D , Teo Y, Boukari H et al Ribosomal mistranslation leads to silencing of the unfolded protein response and increased mitochondrial biogenesis. Commun Biol 2019;2:381. 31637312 10.1038/s42003-019-0626-9 PMC6797716

