# Supplementary figures and images for: Development of a highly sensitive method to detect translational infidelity
Source: Biol Methods Protoc. 2025 Jan 25;10(1):bpaf008. doi: 10.1093/biomethods/bpaf008 (PMC11805343; doi:10.1093/biomethods/bpaf008)

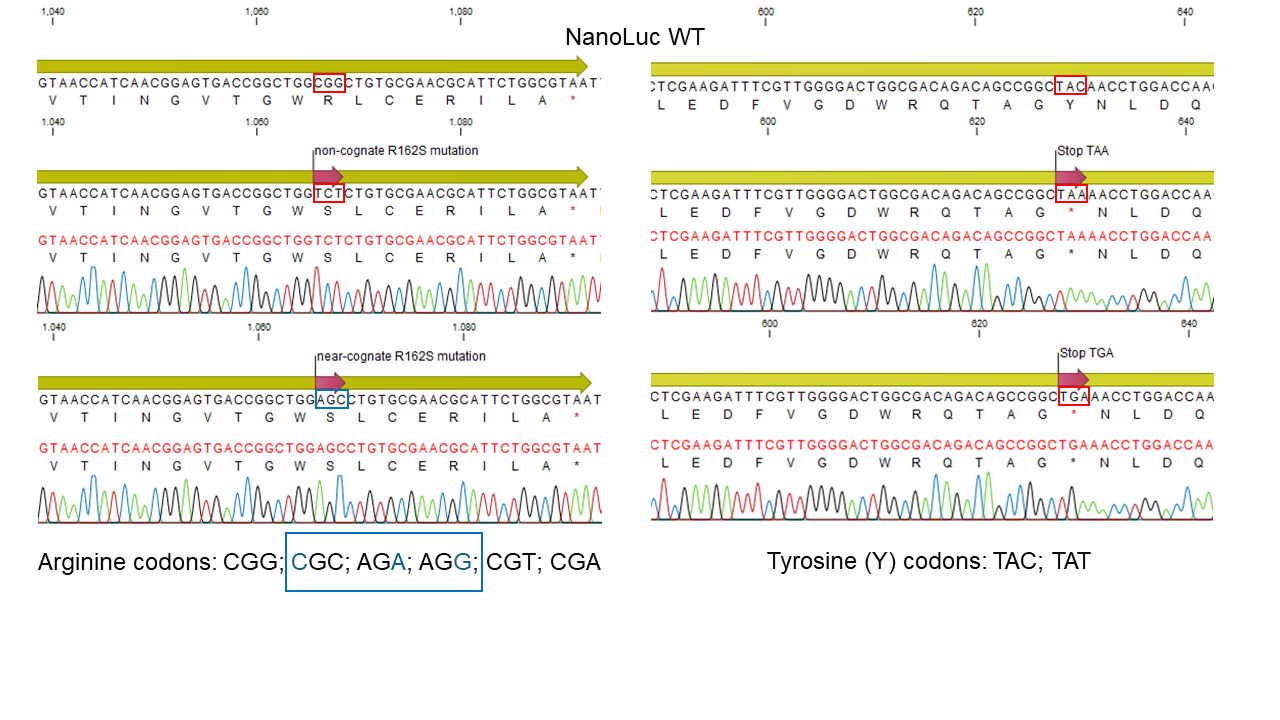

Supplement: bpaf008_Supplementary_Data [file bpaf008_supplementary_data.zip › 78baa_Figure S1 300.jpg]

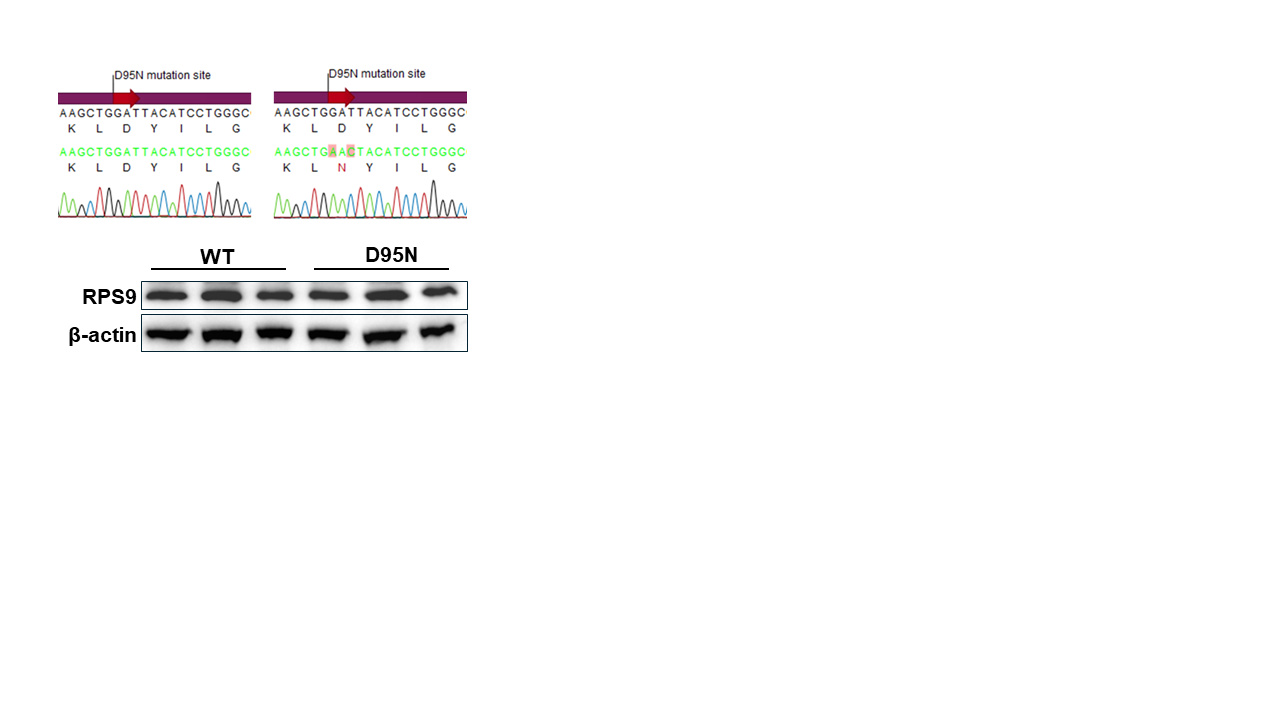

Supplement: bpaf008_Supplementary_Data [file bpaf008_supplementary_data.zip › 6cc41_Figure S2 300.jpg]
